# C-type lectin receptor signaling in schistosomiasis

**DOI:** 10.3389/fimmu.2026.1816742

**Published:** 2026-05-28

**Authors:** Santoshi Chaudhary, Parisa Kalantari

**Affiliations:** Department of Veterinary and Biomedical Sciences, Center for Molecular Immunology, The Pennsylvania State University, University Park, PA, United States

**Keywords:** C-type lectin receptors, immune signaling, schistosome glycans, schistosomiasis, Th17, Th2

## Abstract

Schistosomiasis is a major neglected tropical disease caused by helminth parasites of the genus *Schistosoma*, affecting over 250 million people worldwide and leading to substantial morbidity primarily driven by egg-induced immunopathology. Schistosome parasite can modulate host immunity through stage-specific glycans displayed on cercariae, schistosomula, adult worms, and eggs. These glycans form a critical molecular interface with host C-type lectin receptors (CLRs), a family of carbohydrate-recognition receptors predominantly expressed by dendritic cells, macrophages, and other innate immune cells. CLRs recognize schistosome-derived glycoproteins and glycolipids through C-type lectin-like domains and translate extracellular glycan sensing into intracellular signaling pathways that shape downstream immune responses. Emerging evidence demonstrates that key CLRs, including the mannose receptor, DC-SIGN, MGL, MBL, Dectin-1, Dectin-2, and Mincle, play distinct and sometimes opposing roles during schistosomiasis by regulating antigen uptake, cytokine production, inflammasome activation, and T-helper cell differentiation. Depending on receptor engagement, CLR signaling can promote protective Th2 immunity, drive pathogenic Th17 responses associated with severe hepatic fibrosis, or induce regulatory pathways that limit excessive inflammation. Moreover, CLR-mediated signaling does not occur in isolation but involves extensive crosstalk with other pattern recognition receptors, particularly Toll-like receptors. Despite significant progress, critical gaps remain in understanding receptor specificity, context-dependent signaling, and the role of CLRs in disease susceptibility. This review synthesizes current knowledge on CLR-schistosome interactions, highlighting their central role in immune modulation and disease pathogenesis.

## 1 Introduction

Schistosomiasis is a neglected tropical parasitic disease caused by the trematode helminths of the genus *Schistosoma* (*S.*), affecting 253 million people globally (1, 2). The most common species infecting humans are *S. mansoni*, *S. japonicum*, *S. intercalatum, and S. mekongi*, causing intestinal schistosomiasis*, and S. hematobium*, causing urogenital schistosomiasis (3). According to WHO, *S. mansoni* is of major public concern, being endemic in 55 countries, including most of the cases (around 90%) in Africa, as well as parts of South America, the Caribbeana and Asian countries (4). *Schistosoma* has a complex and indirect life cycle involving two different hosts: mammals serve as the definitive hosts, while a freshwater snail serves as the intermediate host. Eggs hatch into free-swimming miracidia, which penetrate the snail and develop into cercariae- the infective stage. These cercariae penetrate human skin and migrate to the hepatoportal circulation, where they mature into adult worms. The paired male and female worms then migrate to the mesenteric venules, which vary by species. For instance, *S. mansoni* resides mostly in the inferior mesenteric veins draining the large intestine, *S. japonicum* in the superior mesenteric veins draining the small intestine, *S. intercalatum and S. mekongi* in the inferior mesenteric plexus, but lower in the bowel than *S. mansoni*, whereas *S. hematobium* most often inhabits the vesicular and pelvic venous plexus of the bladder. *Schistosoma* begins producing eggs approximately 28 days post-infection (5). The eggs are deposited into the endothelial linings of capillary walls and are either carried to the liver via blood flow or translocated to the intestinal lumen (*S. mansoni*, *S. japonicum, S. mekongi, S. intercalatum)*, bladder, and ureters (*S. hematobium*). Eggs that reach the intestinal lumen are excreted in the feces, and those from the bladder or ureter through urine, completing the parasite’s life cycle (6).

Schistosomiasis varies widely in severity, ranging from mild to severe forms. Mild clinical symptoms include urticarial rash, localized maculopapular lesions, epidermal pustules, dermatitis, fever, cough, headache, abdominal tenderness, and diarrhea. Severe immunopathology occurs in approximately 5-10% of cases and often leads to liver fibrosis, portal hypertension, ascites, gastrointestinal hemorrhage, and, in some instances, death (7, 8). Currently, Praziquantel (PZQ) is the only available drug for treating schistosomiasis, which targets adult worms and eliminates them from the body (9). As the cercariae penetrate the body and transform into schistosomula (cercaria after losing its tail) within the host circulation, a type 1 immune response is elicited, which lasts for about five weeks (10). The elicited immune response is characterized by an increase in interleukin (IL)-1β, IL-12, tumor necrosis factor (TNF)-α, and interferon (IFN)-γ levels in response to worm antigens (11). Once the mature parasites begin producing eggs, a type 2-dominant immune response develops. At this stage, IFN-γ levels decrease, indicating a downregulation of T helper (Th) 1 responses, and a transition toward Th2 cell polarization (12, 13). The protective Th2 response is characterized by expansion of Th2 cells, infiltration of eosinophils, and increased IL-4, IL-5, and IL-13 levels (14). IL-23-IL-1β-IL-17 axis drives severe egg-induced immunopathology (15–17). During this time, granulomas form around the eggs trapped in the hepatic or intestinal tissues, which is the hallmark of Schistosomiasis.

The egg itself is a living and biologically active organism capable of manipulating host immunity. The main immunopathology associated with schistosome infection involves granulomatous inflammation and fibrosis around eggs lodged in the liver and intestines (18). During granuloma formation, neutrophils, macrophages, eosinophils, T and B cells are recruited, and hepatic stellate cells (HSCs) are activated (19). These immune cells surround the eggs, leading to fibrosis and collagen deposition by activated cells, ultimately resulting in hepatic and periportal fibrosis and portal hypertension (20). Progressive portal hypertension worsens the clinical course and can give rise to hepatopulmonary syndrome (HPS), characterized by pulmonary vasodilation and increased intrapulmonary shunting (21). This impairs oxygen exchange, resulting in chronic hypoxia and contributing to poor prognosis and increased patient mortality.

Beyond driving granuloma formation, schistosome egg secretions, including alpha-1 (α-1), omega-1 (ω-1), and eggshell-derived molecules, serve as potent immunomodulators that actively shape host immunity (22). During egg development, fucosylated N- and O-glycans increase in complexity, with highly fucosylated lipid glycans enriched in mature eggs. As the parasite develops, its glycan profiles change markedly across developmental stages. After cercariae-to-schistosomula transformation, N-glycans with Lewis X (LeX) and core-xylose motifs are rapidly lost, while GalNAcβ1–4GlcNAc (LDN) motifs and multifucosylated LDN glycolipids become dominant in adult worms, accompanied by a rapid loss of complex O-glycans (23). Together, these stage-specific glycoproteins and glycolipids form a direct molecular interface between schistosome glycobiology and C-type Lectin Receptor (CLR)-mediated modulation of inflammatory immune responses (24, 25). Schistosome antigens are recognized by the CLRs such as Dectin-1/2 (26), Mannose receptor (MR, CD206) (27), Dendritic cell-specific ICAM-3 grabbing non-integrin (DC-SIGN, CD209) (17) via C-type lectin-like domains (CTLDs). C-type lectins (CTLs) comprise the calcium-dependent glycan-binding proteins that share conserved features within their carbohydrate recognition domain (CRD). This domain is broadly termed the CTLD, as not all CTLD-containing proteins bind to glycan or require calcium for ligand recognition (28). The glycan recognition by many CLRs trigger intracellular signaling motifs that activate Spleen tyrosine kinase (Syk) and Caspase recruitment domain family member 9 (CARD9)-dependent pathways, modulate cytokine production, regulate antigen uptake and presentation, and influence the differentiation of T-helper cells. This makes them uniquely poised to shape the outcome of helminth infections, which rely heavily on glycan-mediated host modulation (29).

Despite significant advances, critical gaps remain in our understanding of CLR-mediated immunity in schistosomiasis. Most studies have focused on a limited subset of receptors or specific schistosome life stages. The downstream intracellular pathways, crosstalk between CLRs and other PRRs, and the role of CLRs in disease susceptibility in human populations remain largely unexplored. Furthermore, schistosome-mediated modulation of CLR signaling represents a powerful immune evasion strategy that is only beginning to be understood. This review synthesizes current knowledge on the role of CLRs in schistosomiasis, with a focus on receptor specificity, signaling mechanisms, and immunological outcomes. By highlighting emerging themes and identifying key gaps, we aim to provide a framework for future studies that will advance our understanding of host-parasite interactions and support the development of CLR-targeted vaccines and therapeutics.

## 2 C- type lectin receptors in schistosomiasis

Pattern recognition receptors (PRRs) interact with pathogen-associated molecular patterns (PAMPs) to initiate innate immune response, shaping subsequent adaptive immunity. PRRs are membrane-bound, like toll-like receptors (TLRs) and CLRs, and cytosolic, including nucleotide-binding oligomerization domain (NOD)-like receptors (NLRs), absent in melanoma 2 (AIM2)-like receptors (ALRs), retinoic acid-inducible gene I (RIG-I), and other DNA and RNA sensors, like cyclic guanosine monophosphates (GMP)-adenosine monophosphates (AMP) synthase (cGAS) (30–32). While TLRs and NLRs have been studied in schistosomiasis, emerging evidence highlights an equally critical yet understudied family of CLRs. CLRs comprise a diverse family of carbohydrate-binding receptors that are predominantly expressed by dendritic cells, macrophages, monocytes, and certain endothelial and epithelial cells (32). These receptors could be either soluble or transmembrane proteins. Soluble CLRs are secreted proteins in serum or at mucosal surfaces, where they function as soluble sensors of PAMPs, such as mannose-binding lectin (MBL). In contrast, transmembrane CLRs contain at least one CTLD, which may function as either a Ca^2+^-dependent CRD or a Ca^2+^-independent lectin-like domain (28). Based on the number of CTLDs, CLRs are broadly classified into two groups. Group I includes the mannose receptor family, whereas group II belongs to the asialogycoprotein receptor family and includes CLRs such as Dectin-1, Dectin-2, Mincle, MGL, and DC-SIGN (25, 33, 34). Recent studies have demonstrated the central role of CLRs in recognizing schistosome-derived antigens, initiating innate immune signaling pathways (**Table 1**), and directing adaptive immune responses. Therefore, the following sections will focus on CLRs and their specific roles during schistosomiasis.

**Table 1:** Gene names, common names, and other protein names of the CLRs discussed in this review, along with their major signaling pathways activated upon recognition of glycans from different developmental stages of Schistosoma spp.

Approved human gene name	Approved mouse gene name	Common name of proteins	Signaling pathways	Ligands	Schistosoma stage
CD209	Cd209a	DC-SIGN (SIGN-R5)	Raf-1	glycolipid-glycans	Schistosomula (35), adult worm (36), SEA (37), eggs (17)
CLEC4E	Clec4e	MINCLE	SYK		Eggs (17, 38)
CLEC6A	Clec4n	DECTIN-2	SYK	α-mannose, PGE2	SEA (26, 39), Eggs (17, 40)
CLEC7A	Clec7a	DECTIN-1	SYK	Omega-1, PGE2	SEA (26)
CLEC10A	Clec10a	MGL	Raf-1	GalNAcB1-4GlcNAc (LDN)	Adult worm (35)
MRC1	Mrc1	MANNOSE RECEPTOR (CD206)		Omega-1	SEA (41), Omega-1 (42) E/S-Schistosomula (27), Adult worm (43)

### 2.1 Mannose-binding lectin

Mannose-binding lectin (MBL) is a soluble CLR that recognizes carbohydrates such as N-acetylglucosamine and N-acetylgalactosamine present on cercariae and adult worms (44, 45). In humans, MBL is encoded by the MBL2 gene, and three single-nucleotide polymorphisms (SNPs) located in exon 1 disrupt MBL oligomer formation, leading to impaired functional activity and reduced circulating serum levels (46). Serum MBL levels have been reported to be inversely associated with susceptibility to *S. haematobium* infection in studies involving infected and non-infected individuals in Africa (47). MBL and L-ficolin, in association with the MBL-associated serine proteases (MASP1 and MASP2), form the complement lectin-cascade, which contributes to pathogen clearance through opsonization (48). L-ficolin is a pattern recognition protein encoded by the FCN2 gene. The FCN2 -986A and -4G alleles are associated with the occurrence of schistosomiasis, where heterozygous genotypes increase the risk of infection, and homozygous allele genotypes appear to function as a protective shield against the disease (44). Furthermore, a study conducted in patients from Zimbabwe showed that higher plasma MBL levels and MBL2 promoter genotypes LY and LL were associated with increased susceptibility to both *S. mansoni* and *S. hematobium* infections (42). Overall, these findings suggest that polymorphism in MBL genes can influence the host response to schistosomiasis.

### 2.2 Mannose receptor

The mannose receptor (MR, CD206) is a type I transmembrane C-type lectin receptor predominantly expressed on macrophages, dendritic cells (DCs), and certain endothelial cells. Structurally, MR comprises an N-terminal cysteine-rich domain, a fibronectin type II domain, and multiple CTLDs that enable recognition and endocytosis of glycosylated ligands bearing mannose, fucose, or N-acetylglucosamine residues. MR shows a preference to Manα1-3/6Man-R compared to other mannose residues and fucosylated ligands (49). Through ligand binding and internalization, MR plays a key role in antigen uptake, immune modulation, and the promotion of anti-inflammatory or tolerogenic immune responses. The egg excretory and secretory (E/S) material released during the transformation of cercaria to schistosomula acts as a ligand for the MR, leading to elevated IFNγ and decreased IL-4 production in skin-draining lymph nodes (27). In contrast, schistosome egg-derived molecules exploit MR-mediated pathways to skew host immunity toward a Th2 and regulatory phenotype (50). MR expression in macrophages in the granulomas increased with IL-4 during *S. mansoni* infection (51). LPS-activated monocytes cultured in the presence of IPSE/α1-stimulated basophils exhibited elevated expression of CD206 and DC-SIGN, accompanied by suppression of proinflammatory cytokine release, including IL-1β, IL-6, and TNF-α, through basophil-derived IL-4 and IL-13 (52). Similarly, studies using site-directed mutagenesis of the *S. mansoni* egg antigen ω1 demonstrated that both its glycosylation and RNase activity are essential for its Th2-inducing capacity *in vitro* and *in vivo*. Mechanistically, ω1 binds to DCs and is internalized via its glycan moieties through MR (45), highlighting the importance of MR-dependent antigen uptake in helminth-driven immune modulation.

The ω1 homolog of *S. japonicum*, SjCP1412, shares limited amino acid identity with *S. mansoni* ω1 yet retains RNase T2 activity and is released exclusively from parasite eggs. Recombinant SjCP1412 induces an alternatively activated (M2) macrophage phenotype characterized by increased expression of CD206, arginase-1, and IL-10, while inhibiting LPS-driven DC maturation (53). *In vivo*, administration of rSjCP1412 reduces IFN-γ levels while elevating IL-4, TGF-β, and regulatory T cell frequencies, broadly recapitulating the immunomodulatory effects of ω1 (53). Additionally, treatment of murine peritoneal or bone marrow-derived macrophages with schistosomal lipid extracts or purified lysophosphatidylcholine (LPC) promotes an MR-associated M2 profile, marked by increased arginase-1, MR1, Ym1, TGF-β, IL-10, and PGE2 production in a PPAR-γ–dependent manner (43). Soluble egg antigen (SEA) glycans induce the expression of suppressor of cytokine signaling 1 (SOCS1) and SH2-containing protein tyrosine phosphatase-1 (SHP1) levels in human DCs (41). SOCS and SHP-1 are negative regulators of cytokine signaling (54) that can prevent excessive inflammation and cytokine signaling.

### 2.3 DC-SIGN (CD209)

DC-SIGN (CD209) is the human receptor that facilitates the attachment of T cells to dendritic cells and acts as a pathogen recognition receptor (55). DC-SIGNR (DC-SIGN-Related receptor) or L-SIGN, CD209L (Liver/lymph node-SIGN) are the receptors found in the endothelial cells of the liver, lymph nodes, and placenta, and are closely related to DC-SIGN (56). DC-SIGN is a type II transmembrane receptor composed of a short N-terminal cytoplasmic tail, a transmembrane domain, an extracellular stalk (neck), and a C-terminal CRD (57). Unlike humans, mouse DC-SIGN is significantly different, featuring a more expanded family of eight paralogs (SIGNR1-SIGNR8) (58). Although human DC-SIGN signals through its cytoplasmic tail, this domain is highly variable among the murine CD209 family. Only CD209a (SIGNR5), CD209b (SIGNR1), CD209d (SIGNR3), and CD209f (SIGNR8) possess full-length cytoplasmic tails, and among these, only CD209a and CD209b retain a conserved juxtamembrane G-C-x-x-H motif shared across species. This pronounced structural heterogeneity has complicated efforts to identify murine proteins that are true functional counterparts of human DC-SIGN or DC-SIGNR. Nonetheless, based on expression patterns, CD209a most closely resembles human DC-SIGN (59).

Schistosome eggs are enriched in glycoproteins containing fucose and mannose residues, which can be detected by multiple CLRs, including DC-SIGN and DC-SIGNR (60). DC-SIGN on dendritic cells recognizes specific sugars (glycans), particularly Lewis X from schistosomula and the worm (36), and E/S products (61). Schistosomula-derived extracellular vesicles (EVs) display CLR ligands on their surface, primarily composed of oligomannosidic and complex glycan structures. These glycans are characterized by a core α1,6-fucose and one or two antennae consisting of Galβ1–4GlcNAc (lacto-N-acetyllactosamine, LacNAc) and/or Lewis X (LeX). Additional structural features include core-xylose modifications and GalNAcβ1–4GlcNAc (LacDiNAc, LDN) antennae bearing multiple fucose residues (23, 24, 61, 62). DC-Sign internalizes the EV-associated glycolipid-glycans with terminal motifs that contain LeX, Fucα1-3Galβ1-4(Fucα1-3) GlcNAc (pseudo-LewisY (LeY)) and GalNAcβ1-4(Fucα1-3) GlcNAc (LDN-F) (24, 63, 64). Schistosome E/S components containing glycan motifs bind to DC-SIGN in a calcium-dependent manner (62). Consistent with this, DC-SIGN expressed in dendritic cells recognizes *S. mansoni* worm-derived glycolipids, and this interaction has been reported to skew naïve T cells’ response towards a Th1 phenotype (36).

CD209 has been found to have a pro-inflammatory immune response as it is critical for the pathogenic Th17 cell response during schistosomiasis, confirming that CBA mice deficient in CD209a have reduced severity of schistosome egg-induced immunopathology (16). Th17 cell development was associated with the activation of SRC, Raf-1, and ERK1/2, followed by the production of IL-1β and IL-23 as downstream effects of CD209a (16, 17, 33) activation in live eggs stimulated BMDCs. We previously demonstrated that CD209a synergizes with Dectin-2 and Mincle to activate proinflammatory signaling cascades leading to severe Th17 cell-mediated Schistosome egg-induced immunopathology (17). CD209b (SIGNR1) recognizes the glycans from *S. mansoni* SEA and worms *in vitro*; however, SIGNR-deficient mice did not show any change in the immunopathology (37).

Mannosylated and Fucosylated ligands differentially regulate DC-SIGN signaling and consequently shape distinct T helper immune responses (33). Mannosylated ligands promote the assembly of a DC-SIGN-associated Raf-1 signalosome, leading to the Raf-1 activation and enhanced production of proinflammatory cytokines (34). This signaling pathway favors Th1- and Th17-associated inflammatory responses. Alternatively, fucosylated ligands induce dissociation of Raf-1 signalosome, supporting anti-inflammatory and Th2-polarizing cytokine responses (65). Among the nine human and murine DC-SIGN paralogs, only DC-SIGNR, CD209a, and SIGNR8 display a strong preference for mannosylated ligands over fucosylated ligands, suggesting a bias towards proinflammatory Raf-1 mediated signaling. Consistent with this concept, CD209a promotes Raf-1 phosphorylation in *S. mansoni* egg-stimulated CBA-derived BMDCs, resulting in Th17-associated immunopathology in murine schistosomiasis. The similarity between murine CD209a and human DC-SIGN in ligand recognition and downstream Raf-1 signaling suggests that a comparable mechanism may operate in humans during schistosomiasis. Depending on the schistosome-derived glycan composition, human DC-SIGN can differentially regulate the context-dependent Raf-1 signalosome formation and favor either Th1/Th17 responses upon recognition of mannosylated ligands or Th2 with fucosylated ligands. Supporting this concept, human DC-SIGN has been reported to be associated with the severity of disease in many infectious diseases, such as Dengue (66).

### 2.4 MGL (Clec10a)

Macrophage galactose-type lectin (MGL) is the CLR within the human system expressed on macrophages and monocyte-derived DCs, which can recognize terminal N-acetylgalactosamine (GalNAc) residues and GalNAcB1-4GlcNAc (LDN) in helminths and tumors (67, 68). This glycan specificity is recognized by the murine MGL2; in contrast, murine MGL1 recognizes Lewis X (69). The EVs from the adult Schistosome worms have abundant N-glycans containing LDN, which were found to significantly interact with the MGL (35) compared to DC-SIGN, which was found to bind with the EV component from Schistosomula (70). Engagement of human MGL by GalNAc-containing ligands activates the ERK MAPK signaling pathway independently of Raf-1 (71, 72). Activated ERK resulted in enhanced secretion of IL-10 and TNFα. In parallel, MGL stimulation also activates the NF-κB pathway, which is required for the increased production of IL-10 (72). In contrast, ligation of murine MGL1 by the helminth *Taenia crassiceps* initiates a Raf-1-dependent but ERK-independent signaling pathway, leading to the suppression of IL-12 and TNFα secretion (73). Although the *S. mansoni* stimulates the generation of regulatory cells and IL-10 production (74), the exact role of the MGL during schistosomiasis has not been elucidated.

### 2.5 Dectin-2

Dectin-2 is a type II transmembrane CLR that binds to sugar ligands such as α-mannose. Dectin-2 is expressed in DCs, macrophages, neutrophils, and monocytes (75). Structurally, it consists of an N-terminal cytoplasmic tail, a transmembrane domain, a stalk, and an extracellular C-terminal CRD (76, 77). Dectin-2 lacks signaling motifs; therefore, it signals by associating with the common Fc Receptor γ subunit (FcRγ), containing an ITAM (immunoreceptor tyrosine-based activation motif) that interacts with Syk Kinase (77). The existing evidence indicates that the functional outcome of Dectin-2 signaling is highly context-dependent, governed by ligand structure, antigen-presenting cell type, and the surrounding environment. *S. mansoni* SEA, which is enriched in mannose glycans, engages Dectin-2 in complex with FcRγ chain in BMDCs from C57BL/6 mice (39, 40). Under conditions where DCs are primed with TLR2 ligands such as Pam3Cys, pro-IL-1β expression is induced, enabling subsequent inflammasome activation. In this inflammatory context, SEA-mediated Dectin-2 signaling activates Syk kinase, leading to reactive oxygen species generation and potassium efflux, which promote ASC-dependent NLRP3 inflammasome assembly, caspase-1 activation, and cleavage of pro-IL-1β into mature IL-1β (40). This pathway drives pro-inflammatory cytokine production, including IL-1β and pathogenic IL-17, while suppressing IL-4 and protective type I IFN (IFN-I), thereby enhancing inflammation (78). Consistent with this, increased NLRP3 activation in the livers of schistosome-infected mice enhances inflammatory responses and promotes extensive tissue damage, inducing connective tissue proliferation, ultimately leading to liver fibrosis (79–81). Similarly, stimulation of BMDCs with live *S. mansoni* eggs, without prior TLR priming, activates the FcRγ-Syk- CARD9-Bcl10-Malt1 (CBM) signaling axis, further promoting IL-1β production and Th17 responses (17). CBM complex is a ternary complex of CARMA1 or CARD9, BCL10, and MALT1, that acts as a signalosome to mediate NF−κB activation.

In contrast, Dectin-2 signaling also contributes to Th2 polarization. DCs stimulated with SEA, but not live eggs, produce prostaglandin E2 (PGE2) through Syk-ERK (extracellular signal-regulated kinase)-cPLA2 (cytosolic phospholipase A2) pathway involving COX-1 and COX-2 (cyclooxygenase 1 and 2) (26, 82). Notably, high-mannose glycans present in SEA serve as ligands for Dectin-2 and are responsible for initiating this PGE2-dependent Th2 immune response (39). Collectively, these findings highlight the context-dependent functionality of Dectin-2, a receptor prominently expressed on DCs that recognizes high-mannose glycans. By integrating microenvironmental cues, including TLR co-stimulation and downstream NLR activation, Dectin-2 orchestrates both inflammasome-driven Th17 pathology and PGE2-mediated Th2 immune responses in schistosomiasis.

### 2.6 Mincle

Mincle (macrophage inducible C−type lectin, CLEC4E) is a type II transmembrane C−type lectin receptor with a short cytoplasmic tail, a single transmembrane region, and an extracellular CRD. It is expressed on various immune cells, including dendritic cells, macrophages, and neutrophils (83). Mincle has been shown to recognize glycolipids such as trehalose-6,6’-dimycolate (TDM) from *Mycobacterium tuberculosis* (84), and glyceroglycolipids from *Malassezia fungus* (85). In the context of schistosomiasis, Mincle participates in the sensing of *S. mansoni* egg-derived glycans as part of the Dectin−2 family CLR cluster on dendritic cells. Within this cluster, Mincle can form functional complexes and synergize with Dectin−2 and CD209a (17) to mediate immune activation. Schistosome egg antigens are enriched in fucosylated and LacDiNAc−type glycan motifs displayed on glycoproteins and glycolipids, which are compatible with the carbohydrate- and lipid-binding preferences of this receptor family and thus provide potential ligands for Mincle.

Mincle signals through its association with the ITAM-bearing FcRγ chain, which is essential for downstream signaling. Ligand binding leads to phosphorylation of the ITAM motif, recruitment of Syk, and activation of the CARD9-Bcl10-Malt1 complex, resulting in canonical NF−κB activation and production of proinflammatory cytokines (17, 85). During *S. mansoni* infection, this pathway promotes the robust production of IL−1β and IL−23, driving the differentiation of pathogenic Th17 cells, which are associated with severe egg−induced hepatic granulomatous pathology (17, 29). Notably, Mincle expression is reduced in *M. tuberculosis*-stimulated macrophages following IL-4 stimulation and *S. mansoni* infection (38). The experimental exposure to mice with schistosome eggs before *Salmonella typhimurium* infection was shown to downregulate Th17 responses in the gut mucosa, thereby impairing protective Th1/Th17-mediated immunity (86). Because the Salmonella cell wall contains trehalose phospholipids that bind to and activate Mincle (87), it is plausible that the induction of IL-17-producing cells during infection requires Mincle signaling, which is inhibited by schistosome eggs-induced IL-4 and IL-13. Therefore, these findings demonstrated that Th2 immune responses can suppress Mincle expression and thereby dampen Th17 immunity in secondary infection, even though Mincle signaling itself promotes pathogenic egg-mediated Th17 responses.

### 2.7 Dectin-1

Dendritic cell-associated C-type lectin receptor-1 (Dectin-1) is a type II transmembrane lectin receptor in the CLR family encoded by the *CLEC7A* gene. It is primarily found on the surface of myeloid lineage cells, including macrophages, dendritic cells, neutrophils, and monocytes (88). Structurally, it comprises a short cytoplasmic tail with a single hemi-immunoreceptor tyrosine-based activation-like motif (hemITAM), a transmembrane region, a stalk, and an extracellular CTLD. The CTLD recognizes glycans, such as β-glucan, which are abundant in fungal cell walls and are critical for antifungal immune response (89). Upon ligand binding, the hemITAM becomes phosphorylated, leading to the activation of Syk or Raf-1, which triggers downstream signaling pathways and activates NF-κB. NF-kB translocates to the nucleus and promotes the production of pro-inflammatory cytokines such as TNF-α, IL-6, IL-23, IL-12, and IL-1β (90). Dectin-1 is shown to be involved in the activation of the inflammasome during *Candida albicans* and *Apergillus fumigates* infections (91, 92). Like the fungal infection, SEA from *S. japonicum* triggered Syk phosphorylation as a downstream event to Dectin-1 in HSCs, leading to NLRP3 inflammasome activation, Caspase 1cleavage, and subsequent IL-1β production (93, 94). *In vivo* studies further demonstrated that mice infected with *S. japonicum* cercaria exhibit increased expression of collagen I and metalloproteinase inhibitor-1 precursor (TIMP), resulting in severe hepatic fibrosis. Moreover, primary macrophages stimulated with SEA or Omega-1 from *S. mansoni* produce IL-1β in a Dectin-1-dependent manner (95).

In contrast to its inflammatory role, Dectin-1 is found to be involved in driving Th2 immune response during schistosomiasis. Stimulation of BMDCs with *S. mansoni* SEA induces Th2 polarization in a PGE2-dependent manner (26), marked by elevated IL-4, IL-5, and IL-13 secretion by T cells. Omega-1 and high-mannose glycans from the SEA do not appear to induce a Th2 immune response through Dectin-1 (39). Overall, while the role of Dectin-1 in fungal immunity is well established, its function in schistosomiasis appears to be complex and context-dependent, involving both inflammasome-driven inflammation and PGE2-mediated Th2 immunity. Nevertheless, the identity of schistosome-derived Dectin-1 ligands and the cell-specific mechanisms governing these divergent responses require further investigation.

## 3 Interaction between CLRs and cell surface PRRs during schistosomiasis

Multiple PRRs, including CLRs and TLRs, recognize distinct antigens derived from cercariae, schistosomula, adult worms, and eggs. As a result, the host’s immune response is likely driven by combined CLR signaling or collaborative interactions between CLRs and TLRs. TLRs comprise a family of 13 paralogs, with 10 expressed in humans and 12 in mice (96). These receptors are either transmembrane receptors located on the cell membrane (TLR1, 2, 4, 5) or within intracellular organelles (TLR3, 7, 8, and 9). TLRs promote innate and adaptive immune responses against pathogens (97). TLRs expressed on skin-resident cells, such as Langerhans cells and mast cells, recognize schistosome cercariae during skin penetration (98). TLR4 is activated by the cercarial E/S products, inducing the production of cytokines like IL-12p40 and IL-10 via the MyD88-dependent signaling pathway (99). Additionally, the schistosomula tegument stimulates the production of IL-12 and TNF-α through TLR4-MyD88 (100, 101). TLR2 recognizes lysophosphatidylserine-containing antigens from the schistosome eggs, promoting Th2 and regulatory T cell differentiation in DCs (102, 103). Furthermore, hepatic TLR3 expression in both myeloid and lymphoid cells is upregulated in mice infected with *S. japonicum*. TLR3-deficient mice exhibit reduced granuloma formation and attenuated liver immunopathology (104). Ritter et al. (40) demonstrated Schistosome SEA suppresses TLR-triggered TNF-α and IL-6 production while enhancing IL-1β secretion in DCs. The production of IL-1β involves NLRP3 inflammasome and Caspase-1 activation (40). The SEA-derived glycan Lacto-N-Fucopentaose III (LNFPIII) interacts with TLR4, activating ERK and mitogen-activated protein kinases (MAPK) signaling in DCs, leading to a Th2-biased immune response characterized by IL-4 production (101, 105).

Importantly, TLR-mediated immune modulation does not occur in isolation but is influenced by interactions with other PRRs, particularly CLRs (106). *S. mansoni* worm glycolipids containing Lewis X motifs are recognized by DC-SIGN, which subsequently cooperates with TLR4 for DC maturation (36). Both DC-SIGN and TLR4 localize within lipid rafts in macrophages following oxidized low-density lipoprotein (oxLDL) stimulation (107), suggesting spatial coordination of signaling. Furthermore, TLR4 activation via a COX-2/PGE2-dependent axis induces HSCs to transdifferentiate, contributing to liver fibrosis during *S. japonicum* infection (108). Furthermore, PGE2 production is induced by the Dectin1/2, promoting Th2 immune responses during *S. mansoni* infection (26), suggesting potential convergence with TLR4-mediated pathways. Additionally, CLRs can collaborate with one another; for instance, CD209a in combination with Dectin-2 and Mincle drives Th17 differentiation and induces severe hepatic immunopathology during *S. mansoni* infection (17).

## 4 Conclusion

**Figure 1:**
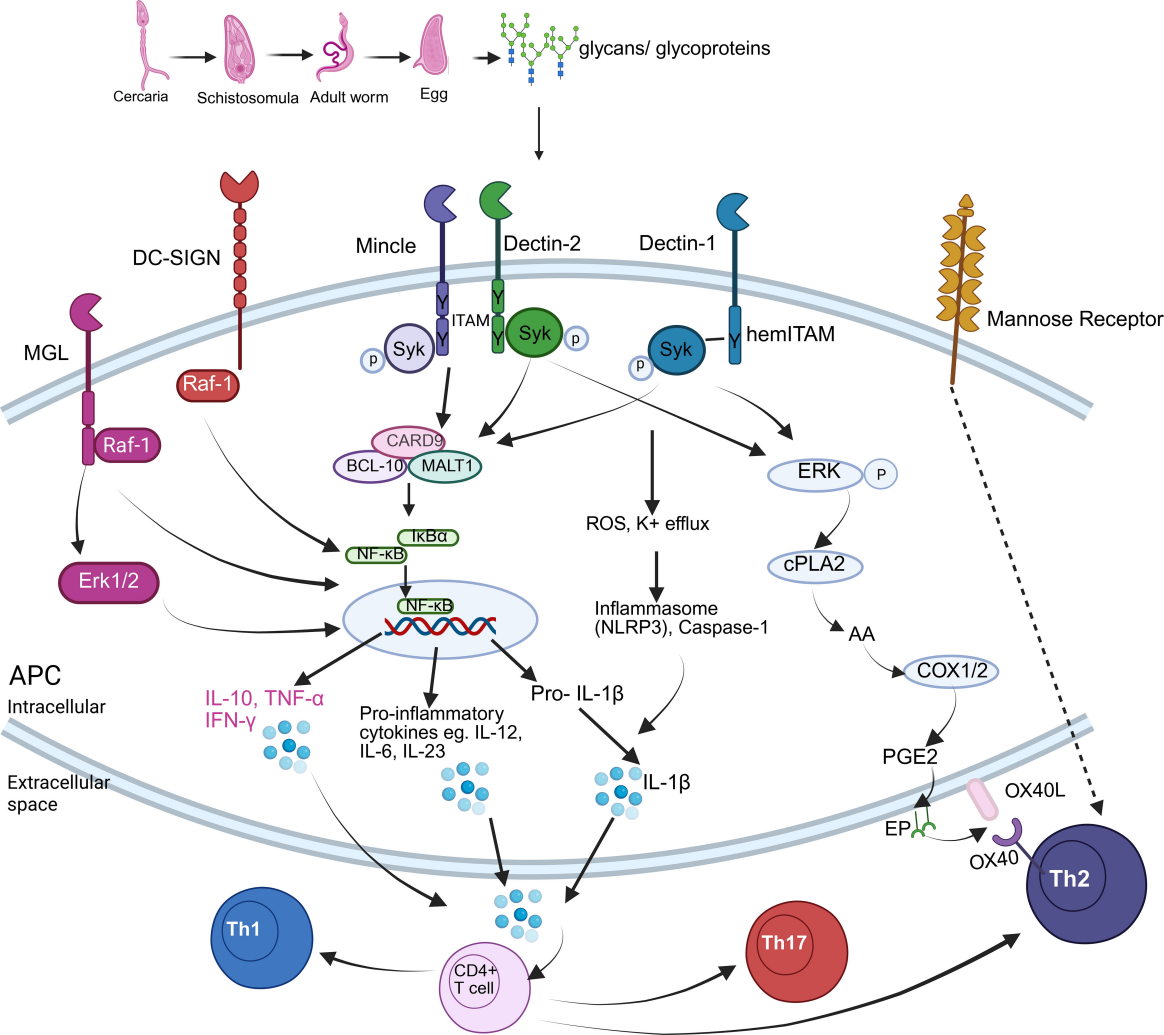
Recognition of Schistosoma spp. glycans by CLRs on antigen-presenting cells (APCs) and downstream signaling pathways. Glycans, glycoproteins, and glycolipids are expressed at different developmental stages of Schistosoma spp. (cercariae, schistosomula, adult worms, and eggs) are recognized by CLRs, including MGL, DC-SIGN, Mincle, Dectin-1, Dectin-2, and the mannose receptor. Ligand engagement activates distinct intracellular signaling pathways, such as Syk-dependent ITAM/hemITAM signaling and Raf-1, leading to activation of NF-κB, ERK1/2, and CARD9–BCL10–MALT1 pathways. These cascades promote production of pro-inflammatory cytokines (e.g., IL-12, IL-6, IL-23, IL-1β), regulatory cytokines (e.g., IL-10), inflammasome activation (NLRP3, caspase-1), and IL-1β maturation, as well as cPLA2-COX1/2-mediated PGE2 synthesis. Collectively, these signaling events shape CD4^+^ T cell polarization toward Th1, Th2, or Th17 responses during schistosome infection.

Schistosomes express a rich array of fucosylated, galactosylated, and trematode-specific glycans on the surfaces of cercariae, schistosomula, adult worms, and eggs. In addition to surface expression, they actively secrete glycan-containing molecules and glycoproteins into the host environment. These glycans, including Lewis X, LDN, LDN-F, LNFP, and multiple glycoproteins such as ω1 and IPSE/α-1interact directly with host CLRs. Through these interactions, CLRs not only mediate antigen recognition and uptake but also drive distinct immunological outcomes ranging from Th1, Th17, Th2, to regulatory immunity (**Figure 1**). Therefore, understanding how different CLRs engage schistosome glycans is fundamental to explaining both protective and pathogenic immune responses.

Research in schistosomiasis remains highly fragmented, with several key questions remaining unresolved. First, it is unclear how individual CLRs integrate signaling when multiple receptors are engaged simultaneously in different stages of schistosome during infection; and second, the cell type-specific expression of CLRs and distribution within tissues such as skin, liver, and lymphoid organs require further investigation to better define downstream signaling pathways and to distinguish protective vs pathogenic CLR-mediated responses. To address these limitations, future research should adopt integrated approaches to map CLR-glycan interactions *in vivo*. Given that glycan profiles shift dramatically throughout the parasite life cycle, how the parasite orchestrates these changes to evade or subvert CLR signaling to facilitate immune evasion remains poorly understood. Therefore, functional studies examining CLRs’ engagement across different life stages of the parasite are needed. The use of advanced and humanized mouse models will also be essential to dissect the receptor-specific contributions of CLRs in this process. A deeper understanding of CLR-mediated parasite-derived glycans will be essential for identifying novel therapeutic targets and advancing intervention strategies.
